# The *Community Eye Health Journal* in your pocket

**Published:** 2019-12-17

**Authors:** Elmien Wolvaardt

**Affiliations:** 1 Editor: *Community Eye Health Journal*, International Centre for Eye Health, London School of Hygiene & Tropical Medicine, UK.

**Access to practical, peer-reviewed and relevant eye health articles at your fingertips – even when internet connection is a challenge.**

The *Community Eye Health Journal* has come a long way in the last three decades: our paper editions reach over twenty thousand people worldwide, our online readership has grown to 120,000 and our articles have been viewed or downloaded on PubMed 1.2 million times.

**Thank you** to Tijssen Foundation and the Peek Vision Foundation (**www.peekvision.org**) for funding the development of the *Community Eye Health Journal* app.

With the rapid increase in cellphone/mobile phone use worldwide, and based on your feedback to our 2010 and 2015 reader surveys, we have now launched the *Community Eye Health Journal* app for Android phones, iPhones, tablets and internet browsers. If you've ever wished you had a copy of the journal with you, or found an article you were in a hurry to share with your colleagues, our new app is just what you need!

## Features

The *Community Eye Health Journal* app allows you to:

- Get access to issues from the last 10 years.
- Find content by year in the **Issues** tab (see the menu bar at the bottom of the screen). Swipe left or right across the top to scroll between years, and tap the year to see the list of issues.
- Look in the **Topic** tab for articles related to common keywords.
- Use the **search tool** (the magnifying glass, top right on most screens) to find something specific.
- Move between the articles in an issue using the **PREV** (previous) and **NEXT** buttons at the top of each article.
- Visit **News** for information about courses, conferences and updates (you will also receive a push notification when a new issue or article is published).
- **Bookmark** or **download** articles and issues when you're online.
- Find and organise your bookmarks and downloads in your own personal **Library**.

**Figure:**
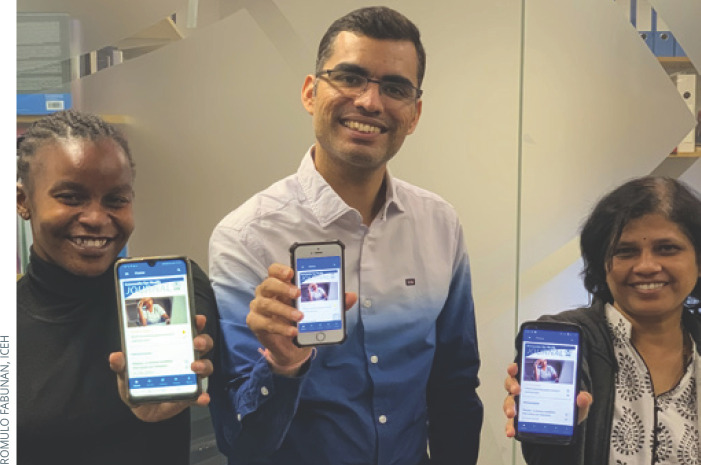
Ophthalmologists from Kenya and India studying at the London School of Hygiene & Tropical Medicine.

**Figure 1:**
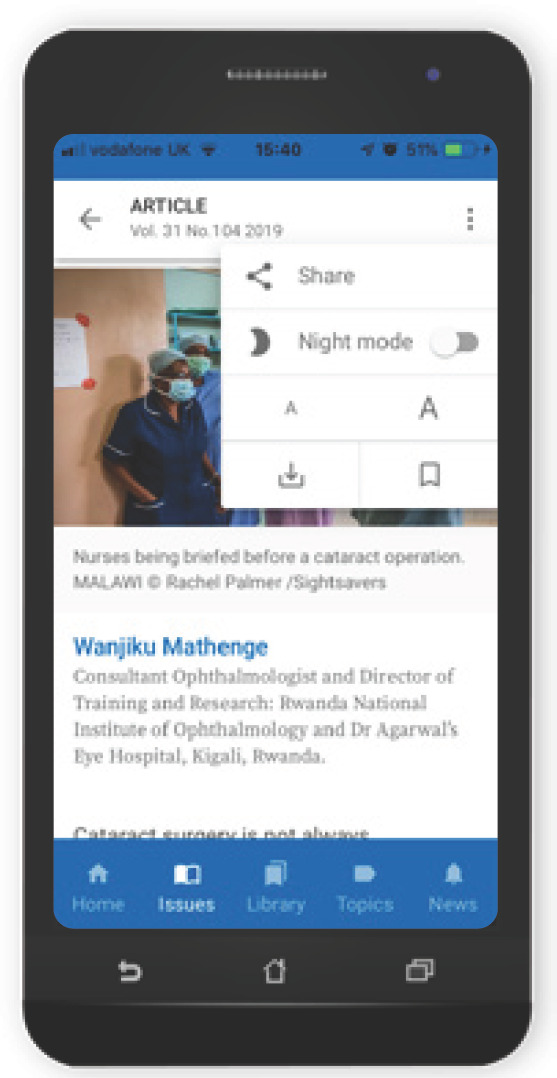
The three dots top right open up a menu where you can share, improve visibility and download or bookmark articles.

When you read an article, you will find even more useful features in the pop-up menu – tap the three dots top right to view it (Figure 1). Here you can:

- **Share** the article via email or social media
- Select **Night mode** (white text on a black background) to improve contrast
- **Change the text size** to improve visibility.
- **Bookmark** or **download** the article to the Library

To save any image to your camera roll, tap and hold the image until the option appears.

## Library

Here you can create and name your own folders to organise your content, e.g. for outreach visits, teaching or patient education. To access your library, you will be asked to create a free and secure account using an email address and a password of your own choosing. Everything you have downloaded and organised in your folders will always be accessible, even if you change devices or lose a device. You can also log into your library account on multiple devices, including your mobile, tablet or computer.

## We want to hear from you!

We have worked hard to make the app as useful, user-friendly and accessible as possible. Have we succeeded? Is there anything else you would like the app to be able to do? Get in touch via Twitter or Facebook (@CEHJournal) or email us at **admin@cehjournal.org** Let us know if you notice any errors or need help using the app.

## Download the app

Find our app in **Google Play** (Android) or the **App Store** (iOS): search for **‘Community Eye Health Journal’** or **‘CEHJ’**.

**Figure:**
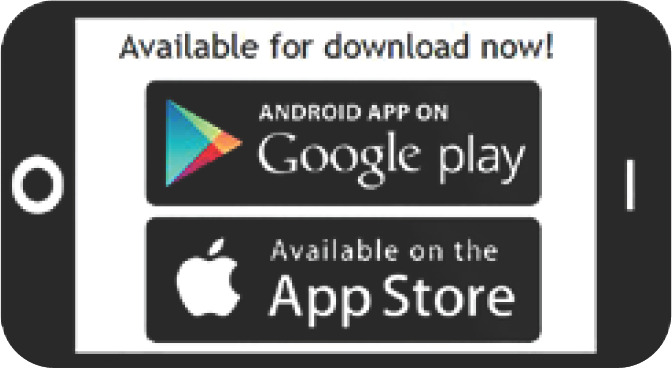


You can also view the app in any browser, on any device, by visiting **https://m.cehjournal.org**

